# A new dyslipidemia-based scoring model to predict transplant-free survival in patients with hepatitis E-triggered acute-on-chronic liver failure

**DOI:** 10.1186/s12944-023-01826-y

**Published:** 2023-06-24

**Authors:** Chong Chen, Aihong Zhu, Shanke Ye, Weixia Li, Ling Fei, Qin Huang, Liang Chen

**Affiliations:** 1 Department of Infectious Diseases, Shanghai Public Health Clinical Center, Fudan University, 2901 Caolang Road, Jin-Shan District, Shanghai, 201508 China; 2 Department of Hepatology, Shanghai Public Health Clinical Center, Fudan University, 2901 Caolang Road, Jin-Shan District, Shanghai, 201508 China

**Keywords:** Hepatitis E virus, Acute-on-chronic liver failure, Triacylglycerol, apoA, Prognostic scoring model

## Abstract

**Background/Aims:**

Hepatitis E virus (HEV)-triggered acute-on-chronic liver failure (ACLF) has unacceptably high short-term mortality. However, it is unclear whether the existing predictive scoring models are applicable to evaluate the prognosis of HEV-triggered ACLF.

**Methods:**

We screened datasets of patients with HEV-triggered ACLF from a regional tertiary hospital for infectious diseases in Shanghai, China, between January 2011 and January 2021. Clinical and laboratory parameters were recorded and compared to determine a variety of short-term mortality risk factors, which were used to develop and validate a new prognostic scoring model.

**Results:**

Out of 4952 HEV-infected patients, 817 patients with underlying chronic liver disease were enrolled in this study. Among these, 371 patients with HEV-triggered ACLF were identified and allocated to the training set (n = 254) and test set (n = 117). The analysis revealed that hepatic encephalopathy (HE), ascites, triacylglycerol and apolipoprotein A (apoA) were associated with 90-day mortality (*P* < 0.05). Based on these significant indicators, we designed and calculated a new prognostic score = 0.632 × (ascites: no, 1 point; mild to moderate, 2 points; severe, 3 points) + 0.865 × (HE: no, 1 point; grade 1–2, 2 points; grade 3–4, 3 points) − 0.413 × triacylglycerol (mmol/L) − 2.171 × apoA (g/L). Compared to four well-known prognostic models (MELD score, CTP score, CLIF-C OFs and CLIF-C ACLFs), the new scoring model is more accurate, with the highest auROCs of 0.878 and 0.896, respectively, to predict 28- and 90-day transplantation-free survival from HEV-triggered ACLF. When our model was compared to COSSH ACLF IIs, there was no significant difference. The test data also demonstrated good concordance.

**Conclusions:**

This study is one of the first to address the correlation between hepatitis E and serum lipids and provides a new simple and efficient prognostic scoring model for HEV-triggered ACLF.

**Supplementary Information:**

The online version contains supplementary material available at 10.1186/s12944-023-01826-y.

## Introduction

Patients with chronic liver diseases (CLDs) may develop acute-on-chronic liver failure (ACLF), which is typically triggered by a precipitating event and represents a common route to death [1, 2]. The consensus from the Asian Pacific Association for the Study of the Liver (APASL) and recent epidemiological surveys suggested that the prevalence of hepatitis E virus (HEV) infection could act as one of the most common triggers for ACLF [3–5]. Moreover, patients with HEV-triggered ACLF are more likely to have worse outcomes, with an unacceptably high short-term mortality rate ranging from 10 to 40% [5, 6]. Several large multicenter prospective studies have documented the clinical features of ACLF, such as an intense systemic inflammatory response, which progresses to multiple organ failure and ultimately death [1, 2, 7]. Given the intractable condition and high mortality rate of ACLF, a variety of noninvasive models based on laboratory parameters, liver complications and organ functions have been developed to assess the severity of ACLF and prognosis. For instance, typical scoring models from the Chinese Group on the Study of Severe Hepatitis B (COSSH) and Chronic Liver Failure-Consortium (CLIF-C), as well as the relatively earlier Model for End-Stage Liver Disease (MELD) score and Child-Turcotte-Pugh (CTP) score, showed high diagnostic sensitivity and prognostic accuracy for hepatitis B-triggered ACLF, cirrhosis-related ACLF and alcohol liver disease-related ACLF [2, 8, 9]. However, the applicability of these aforementioned scoring models is unknown for HEV-triggered ACLF. In particular, the pathogenic process of ACLF triggered by hepatitis E compared to other precipitating etiologies may be different and has not been investigated in depth to date.

As an essential organ of metabolism, the liver plays a major role in regulating lipid metabolism [10, 11]. Previous studies have shown that hepatitis virus infection may interfere with lipid metabolism and even act as a major factor in hepatic steatosis in infected patients [12–14]. Meanwhile, a wide range of metabolic abnormalities derived from severe liver damage, especially lipid metabolic disorder, has been reported to be associated with adverse outcomes in hepatitis B virus (HBV) and hepatitis C virus (HCV) infections and ACLF [12, 14, 15]. It is worth noting that researchers have suggested that targeting lipid metabolism could be an antiviral strategy against HEV [16]. Cellular lipid metabolism is also associated with viral replication and the assembly of HEV virions [17, 18]. When severe liver damage is caused by HEV infection, a range of changes may occur in the intrahepatic and extrahepatic microenvironments [19], causing systemic perturbations. In addition, hyperinflammatory status in ACLF patients with multiple organ failure is associated with variations in lipid molecules [20]. Overall, these studies reinforce the concept that there may be a strong relationship between serum lipids and severe liver damage associated with HEV.

As mentioned above, HEV-triggered ACLF may encompass the process of disrupting lipid metabolism and immune abnormalities. Thus, we hypothesize that serum lipid levels are associated with liver damage and disease manifestation in patients with HEV-triggered ACLF. It is important to note that the components of previous score models do not take into account these features of lipid disorders. In this observational study, we seek to describe the variation in lipid metabolism from different stages of the disease and to analyze their potential links with severe hepatitis and the prognosis of HEV-triggered ACLF, as well as to provide a simpler biomarker and more valid scoring model for predicting the prognosis of HEV-triggered ACLF.

## Methods

### Study design

This 11-year retrospective cohort of HEV-triggered ACLF cases was selected for study in the Shanghai Public Health Clinical Center (the regional tertiary hospital for infectious diseases in Shanghai, China). The inclusion and exclusion criteria for HEV-triggered ACLF are provided as part of online Supplementary File 1. The diagnosis of HEV infection, liver cirrhosis and ACLF, as well as the definition of underlying CLDs, have also been summarized in online Supplementary Files 3 and 4. This study protocol conforms to the ethical guidelines of the 1975 Declaration of Helsinki (6th revision, 2008) and has been approved by the Institutional Review Board of the Shanghai Public Health Clinical Center (NO.2022-S075-01). The requirement for individual written informed consent was waived as the study was retrospective in design, all patient information was anonymous, and only existing data were used.

We collected data on clinical information and laboratory tests from hospital databases or medical records. Demographic data included age, sex and alcohol use. Clinical complications include ascites, hepatic encephalopathy (HE), bacterial infection (BI) and gastrointestinal hemorrhage. Laboratory parameters included liver function, coagulation indicators, serum lipids and other related laboratory results. Patients who received liver transplantation (LT) were excluded. All included patients were followed up for 90 days from the point of admission and received integrative treatment during hospitalization (death was the evaluation endpoint).

### Study procedures

Based on the APASL criteria, our study cohort could be separated into two groups: one with HEV-triggered ACLF and one without HEV-triggered ACLF. The HEV-triggered ACLF group consisted of survivors and nonsurvivors (follow-up time was 90 days after admission). Clinical and laboratory characteristics (including serum lipid levels) of patients were compared between the above two groups to gather preliminary information. Patients with HEV-triggered ACLF were also divided into two groups according to whether they had bacterial infection during the course of the disease. The relationship between lipid metabolism and bacterial infection in HEV-triggered ACLF was investigated by comparing the differences in lipid levels between the above two groups. In addition, the association of organ failure and serum lipids in HEV-triggered ACLFs was analyzed. Organ failure was defined based on Chronic Liver Failure-Sequential Organ Failure Assessment (CLIF-SOFA) diagnostic criteria [9] and evaluated within 90 days of admission.

### Development and validation of a new prognostic score model

Patients with HEV-triggered ACLF were divided into the training set and test set based on admission time. Patients admitted between January 2011 and December 2017 (n = 254) were used to calculate and develop a new scoring model. Univariate analysis was first conducted to examine each of the factors influencing short-term mortality in HEV-triggered ACLF. Multivariate Cox regression analysis was then used to reveal independent factors for 90-day LT-free mortality (Table 1). We integrated all the calculated parameters and incorporated them into a new HEV-triggered ACLF scoring model (for simplicity, this new model has been called HEV-T-ACLFs in the following description). We then evaluated and compared the performance of the new HEV-T-ACLFs model. Subsequently, the external test set was obtained using data collected over a later study period from January 2018 to December 2021 (n = 117). All data required to calculate HEV-T-ACLFs were measured and are presented in Supplementary Table 2 (training set and test set).

**Table 1:** Univariate and multivariate Cox regression analysis of LT-free 90-day mortality in patients with HEV-triggered ACLF.

Parameters	Nonsurvivors (n = 83)	Survivors (n = 171)	*P* value	Training set (n = 254)	Univariate analysis		Multivariate analysis	
					HR (95% CI)	*P* value	HR (95% CI)	*P* value
Age, (y)	55 ± 14	53 ± 13	0.474	53 ± 14	1.006 (0.990–1.021)	0.466		
Gender (male)	70 (84.3)	136 (79.5)	0.397	206 (81.1)	1.255 (0.694–2.268)	0.453		
Alcohol use	31(37.4)	71 (41.5)	0.586	102 (40.2)				
Cirrhosis	58 (69.9)	85 (49.7)	0.003	143 (56.3)	1.908 (1.187–3.067)	0.008	1.708 (0.980–2.976)	0.059
Ascites	49 (59.0)	43 (25.1)	< 0.001	92 (36.2)	2.668 (1.719–4.141)	< 0.001	1.855 (1.012–3.399)	0.046
Bacterial infection	28 (33.7)	32 (18.7)	0.011	60 (23.6)	1.646 (1.044–2.595)	0.032	0.981 (0.560–1.718)	0.947
Hepatic encephalopathy	35 (42.2)	5 (2.9)	< 0.001	40 (15.7)	3.901 (2.523–6.031)	< 0.001	2.027 (1.218–3.373)	0.007
Gastrointestinal haemorrhage	7 (8.4)	6 (3.5)	0.128	13 (5.1)	1.707 (0.787–3.703)	0.176		
ALT, U/L	423 (74-1100)	554 (130–1238)	0.863	526 (119–1211)	NA	0.813		
AST, U/L	155(119–202)	354 (104–955)	0.553	339 (108–990)	NA	0.780		
Tbil, µmol/L	364 (173–495)	223 (130–327)	< 0.001	246 (140–401)	1.002 (1.001–1.002)	< 0.001	1.000 (0.999–1.001)	0.968
Albumin, g/L	32 ± 5	34.5 ± 5	0.002	33 ± 5	0.952 (0.915–0.990)	0.015	1.031 (0.969–1.096)	0.335
INR	2.10 (1.60–2.85)	1.41 (1.15–1.68)	< 0.001	1.57 (1.13–2.10)	1.531 (1.328–1.765)	< 0.001	1.216 (0.978–1.513)	0.079
Creatinine, µmol/L	68.2 (53.3–83.1)	63.1 (53.9–73.3)	0.068	64.3 (53.8–75.1)	1.002 (1.000-1.003)	0.027	1.000 (0.999–1.002)	0.699
Glucose, mmol/L	4.89 (3.96–6.36)	5.08 (4.10–6.41)	0.273	5.07 (4.09–6.39)	0.965 (0.893–1.044)	0.381		
WBC, 10^9^/L	6.90 (5.46–10.44)	5.74 (4.16–7.78)	0.001	6.08 (4.43–8.34)	1.080 (1.027–1.135)	0.003	1.027 (0.965–1.093)	0.404
Haemoglobin, g/L	127 (110–142)	127 (114–146)	0.344	127 (113–144)	0.995 (0.986–1.005)	0.337		
Platelet, 10^9^/L	86 (66–119)	111 (71–153)	0.011	100 (68–146)	0.996 (0.992-1.000)	0.070		
Cholesterol, mmol/L	2.01 (1.39–2.54)	2.96 (2.38–3.72)	< 0.001	2.63 (2.01–3.24)	0.569 (0.447–0.724)	< 0.001	1.310 (0.891–1.928)	0.170
Triacylglycerol, mmol/L	1.22 (0.91–1.63)	1.65 (1.04–2.38)	< 0.001	1.46 (1.00-2.15)	0.611 (0.455–0.820)	0.001	0.551 (0.318–0.955)	0.034
ApoA, g/L	0.15 (0.05–0.27)	0.32 (0.18–0.67)	0.013	0.26 (0.13–0.53)	0.070 (0.022–0.226)	< 0.001	0.200 (0.040–0.991)	0.049
ApoB, g/L	0.66 (044-0.92)	0.91 (0.61–1.23)	0.005	0.80 (0.55–1.14)	0.339 (0.188–0.610)	< 0.001	0.958 (0.638–1.439)	0.837
HDL-C, mmol/L	0.18 (0.11–0.29)	0.20 (0.14–0.55)	0.013	0.19 (0.13–0.40)	0.282 (0.111–0.717)	0.008	0.557 (0.128–2.419)	0.435
LDL-C, mmol/L	0.41 (0.20–1.22)	0.91 (0.25–1.80)	0.005	0.78 (0.24–1.52)	0.699 (0.527–0.926)	0.013	0.714 (0.432–1.179)	0.188

**NOTE**: Data are presented as the median (Q1-Q3), the mean ± SD or the number of patients (%). The categorical data between two groups were compared with chi-square test or Fisher’s exact test. The continuous data between two groups were compared by t test or Mann-Whitney test. Univariate and Multivariate Cox regression models were used to assess the associations between various risk factors and different disease outcomes, as indicated. Nonsurvivors were determined by liver transplantation-free 90-day death.

**Abbreviations**: LT, liver transplantation; HEV, hepatitis E virus; ACLF, acute-on-chronic liver failure; HR, Hazard ratio; CLDs, chronic liver diseases; ALT, alanine aminotransferase; AST, aspartate aminotransferase; ALP, alkaline phosphatase; GGT, gamma-glutamyl transpeptidase; TBA, total bile acid; Tbil, total bilirubin; Dbil, direct bilirubin; PTA, prothrombin activity; INR, international normalized ratio; PT, prothrombin; WBC, white blood cell; ApoA, apolipoprotein A; ApoB, apolipoprotein B; HDL-C, high-density lipoprotein cholesterol; LDL-C, low-density lipoprotein cholesterol; NA, not available.

### Statistical analyses

All statistical analyses in this study were performed using SPSS 26.0 software, GraphPad Prism (GraphPad Software Inc.) and MedCalc Software (MedCalc Software, Belgium). The Kolmogorov‒Smirnov test was used to confirm the normality of the data. Normal continuous variables are presented as the mean ± SD, nonnormal variables as the median and range (Q1-Q3), and categorical variables as the percentage (%). Categorical data between two groups were compared using the chi-square test or Fisher’s exact test. Continuous data between two groups were compared using the t test or Mann‒Whitney test. Multivariate Cox regression analysis was used to examine mortality risk factors, from which the initial model was constructed. The areas under receiver operator characteristic (AUROC) curves were compared to evaluate the predictive value of the different scoring systems. All significance tests were two-tailed, and *P* < 0.05 was considered significantly different between groups.

## Results

### Study subjects

**Fig. 1:**
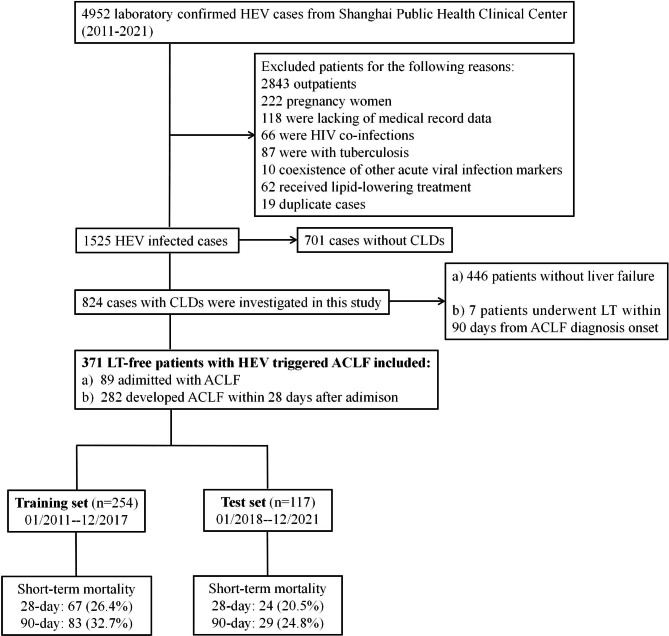
Flow diagram for screening and inclusion of patients studied. In total, 817 HEV-infected patients with underlying chronic liver diseases (CLDs) were included in the study. Of these, 371 patients were diagnosed with ACLF, did not undergo liver transplantation and were assigned to the training (254, 68.5%) and test sets (117, 31.5%) **Abbreviations**: HEV, hepatitis E virus; HIV, human immunodeficiency virus; CLDs, chronic liver diseases; LT, liver transplantation; ACLF, acute-on-chronic liver failure.

A total of 4952 patients were initially screened in this study, and the majority of patients were excluded for a number of reasons (detailed information presented in Fig. 1 and Supplementary File 1). Eventually, 824 patients with HEV infection and underlying CLDs were enrolled in this study. It should be noted that 7 patients who underwent LT within 90 days of the onset of HEV-triggered ACLF were excluded. A total of 371 HEV-triggered ACLF patients (allocated to the training set and test set) were used to develop and validate a new scoring model. Consequently, these patients were followed for 90 days, and their 28-day and 90-day mortality rates were 26.4% and 32.7% in the training set and 20.5% and 24.8% in the test set, respectively. Figure 1 shows the patient screening workflow.

### Clinical characteristics of the enrolled patients

Baseline demographic characteristics, laboratory examination and clinical presentation of the HEV-triggered ACLF patients are summarized in Table 2. The majority of enrolled subjects were middle-aged men (72.9% were male with a median age of 54). The most common underlying CLDs in these patients were chronic hepatitis B (CHB) (60.0%), followed by alcoholic liver disease (18.6%) and fatty liver disease (11.5%). In total, 371 patients (371/817, 45.4%) developed ACLF at enrollment or during the 28-day follow-up period after hospitalization. ACLF patients were older and had a significantly higher percentage of males than non-ACLFs (83.0% vs. 64.6%, *P* < 0.001). The comparison also showed that ACLF patients had significantly higher levels of alanine aminotransferase (ALT), aspartate aminotransferase (AST), total bilirubin (Tbil), and direct bilirubin (Dbil) (all *P* < 0.001) and more serious coagulopathy (with a significantly lower level of prothrombin activity (PTA) and a significantly higher international normalized ratio (INR), both *P* < 0.001) than those without ACLF. Significant differences in white blood cells (WBCs), platelet counts, hemoglobin and levels of various serum lipids were also observed between the two groups. The prevalence of hepatic complications (ascites, BI, HE and gastrointestinal hemorrhage) in ACLF patients was also higher (all *P* < 0.001).

**Table 2:** Comparison of clinical data between patients with or without liver failure, and survival or mortality

Parameters	Total patients (n = 817)	Developed ACLF			LT-free 90-day mortality in ACLF group		
		Yes (n = 371)	No (n = 446)	*P* value	Yes (n = 112)	No (n = 259)	*P* value
Age (y)	54 (42–63)	55 ± 14	51 (37–61)	< 0.001	57 ± 14	55 ± 14	0.351
Gender (male, %)	596 (72.9)	308 (83.0)	288 (64.6)	< 0.001	95 (84.8)	213 (82.2)	0.652
Alcohol user, n (%)	281 (34.4)	159 (42.9)	122 (27.4)	< 0.001	43 (38.4)	116 (44.8)	0.304
Cirrhosis, n (%)	378 (46.3)	191 (51.5)	187 (41.9)	0.007	76 (67.9)	115 (44.4)	< 0.001
Chronic liver diseases, n (%)							
Primary hepatic carcinoma	30 (3.7)	23 (6.2)	7 (1.6)	< 0.001	14 (12.5)	9 (3.5)	0.002
Chronic hepatitis B	490 (60.0)	225 (60.7)	265 (59.4)	0.774	74 (66.1)	151 (58.3)	0.167
Chronic hepatitis C	36 (4.4)	12 (3.2)	24 (5.4)	0.171	4 (3.6)	8 (3.1)	0.759
Fatty liver disease	94 (11.5)	56 (15.1)	38 (8.5)	0.004	13 (11.6)	43 (16.6)	0.269
Alcoholic liver disease	152 (18.6)	77 (20.8)	75 (16.8)	0.176	18 (16.1)	59 (22.8)	0.164
Autoimmune liver disease	34 (4.2)	6 (1.6)	28 (6.3)	0.001	2 (1.8)	4 (1.5)	0.997
Schistosomiasis	18 (2.2)	13 (3.5)	5 (1.1)	0.029	3 (2.7)	10 (3.9)	0.762
Cirrhosis with unknown factor	37 (4.5)	12 (3.2)	25 (5.6)	0.128	3 (2.7)	9 (3.5)	0.998
Clinical complications, n (%)							
Ascites	169 (20.7)	149 (40.2)	20 (4.5)	< 0.001	75 (67.0)	74 (28.6)	< 0.001
Bacterial infection	206 (25.2)	196 (52.8)	10 (2.2)	< 0.001	100 (89.3)	96 (37.1)	< 0.001
Hepatic encephalopathy	49 (6.0)	49 (13.2)	0	< 0.001	42 (37.5)	7 (2.7)	< 0.001
Gastrointestinal haemorrhage	22 (2.7)	20 (5.4)	2 (0.4)	< 0.001	12 (10.7)	8 (3.1)	0.005
Liver function, coagulation indicators and other related laboratory examinations							
ALT, U/L	267 (56-1000)	717 (154–1455)	117 (38–549)	< 0.001	614 (153–1408)	762 (161–1479)	0.356
AST, U/L	158 (56–615)	406 (147–1089)	77 (38–218)	< 0.001	391 (163–1237)	414 (136–1055)	0.810
ALP, U/L	134 (97–181)	154 (122–198)	114 (82–159)	< 0.001	153 (121–196)	155 (122–199)	0.786
GGT, U/L	117 (59–217)	112 (69–195)	120 (51–233)	0.955	99 (57–140)	123 (71–235)	0.003
Cholinesterase, U/L	4701 (3050–6668)	3416 (2354–4709)	6135 (4335–7843)	< 0.001	2737 (1965–3898)	3758 (2537–5018)	< 0.001
TBA, µmol/L	95 (17–179)	169 (112–225)	23 (8–83)	< 0.001	178 (130–232)	166 (105–224)	0.128
Tbil, µmol/L	76 (21–217)	239 (144–373)	23 (14–58)	< 0.001	359 (184–489)	216 (133–313)	< 0.001
Dbil, µmol/L	53 (10–167)	178 (106–275)	12 (6–42)	< 0.001	257 (125–331)	169 (93–230)	< 0.001
Total protein, g/L	66 (61–72)	63 ± 8	69 ± 8	< 0.001	61 ± 8	64 ± 8	< 0.001
Albumin, g/L	36 ± 6	33 ± 5	38 ± 6	< 0.001	31 ± 5	33 ± 5	< 0.001
PTA, %	80 (58–98)	56 (42–78)	92 (78–102)	< 0.001	38 (28–54)	65 (50–84)	< 0.001
INR	1.16 (1.01–1.45)	1.49 (1.17–1.88)	1.06 (0.99–1.17)	< 0.001	2.05 (1.56–2.74)	1.33 (1.11–1.65)	< 0.001
PT, s	15 (13–18)	18 (15–22)	13.8 (13.1–14.9)	< 0.001	23 (19–29)	16 (14–19)	< 0.001
Creatinine, µmol/L	64.0 (54.2–74.8)	65.2 (54.3–77.6)	63.3 (54.2–73.5)	0.067	69.5 (54.1–88.2)	63.3 (54.3–75.0)	0.024
Glucose, mmol/L	5.21 (4.55–6.48)	5.42 (4.39–7.09)	5.16 (4.65–6.14)	0.276	5.39 (4.10–7.04)	5.43 (4.53–7.10)	0.196
WBC, 10^9^/L	5.52 (4.22–7.09)	6.06 (4.56–8.20)	5.11 (4.08–6.40)	< 0.001	6.72 (5.07–10.29)	5.76 (4.35–7.72)	0.001
Haemoglobin, g/L	133 (119–147)	129 (115–145)	135 (121–148)	< 0.001	128 (110–141)	129 (116–146)	0.115
Platelet, 10^9^/L	135 (90–186)	114 (78–163)	151 (104–205)	< 0.001	94 (71–141)	124 (84–169)	0.001
Baseline lipid levels							
Cholesterol, mmol/L	3.44 (2.61–4.27)	2.72 (2.04–3.45)	3.95 (3.32–4.70)	< 0.001	2.03 (1.41–2.63)	2.99 (2.42–3.64)	< 0.001
Triacylglycerol, mmol/L	1.36 (0.91–2.11)	1.58 (1.10–2.38)	1.17 (0.81–1.76)	< 0.001	1.30 (0.99–1.74)	1.81 (1.20–2.66)	< 0.001
ApoA, g/L	0.67 (0.29–1.05)	0.28 (0.16–0.50)	0.97 (0.73–1.23)	< 0.001	0.19 (0.08–0.28)	0.35 (0.21–0.57)	< 0.001
ApoB, g/L	0.85 (0.65–1.15)	0.92 (0.61–1.24)	0.82 (0.66–1.06)	0.018	0.71 (0.49-1.00)	1.00 (0.69–1.34)	< 0.001
HDL-C, mmol/L	0.58 (0.18–1.07)	0.17 (0.13–0.32)	1.01 (0.66–1.31)	< 0.001	0.15 (0.13–0.26)	0.18 (0.13–0.44)	< 0.001
LDL-C, mmol/L	1.85 (1.07–2.63)	1.13 (0.38–1.74)	2.35 (1.78-3.00)	< 0.001	0.77 (0.32–1.47)	1.22 (0.52–1.93)	< 0.001

**NOTE**: Data are presented as the median (Q1-Q3), the mean ± SD or the number of patients (%). The categorical data between two groups were compared with chi-square test or Fisher’s exact test. The continuous data between two groups were compared by t test or Mann-Whitney test.

**Abbreviations**: CLDs, chronic liver diseases; ALT, alanine aminotransferase; AST, aspartate aminotransferase; ALP, alkaline phosphatase; GGT, gamma-glutamyl transpeptidase; TBA, total bile acid; Tbil, total bilirubin; Dbil, direct bilirubin; PTA, prothrombin activity; INR, international normalized ratio; PT, prothrombin; WBC, white blood cell; ApoA, apolipoprotein A; ApoB, apolipoprotein B; HDL-C, high-density lipoprotein cholesterol; LDL-C, low-density lipoprotein cholesterol.

To determine if there was a difference between survivors and nonsurvivors among patients with ACLF, further comparisons between the two groups were examined (shown in the last three columns of Table 2). As expected, more severe liver damage, more severe coagulopathy and more abnormal biochemical indicators (TBA, creatinine and albumin) were observed in nonsurvivors. Surprisingly, the levels of many serum lipids were exceptionally low for nonsurvivors upon admission (cholesterol: 2.03 mmol/L vs. 2.99 mmol/L; triacylglycerol: 1.30 mmol/L vs. 1.81 mmol/L; apolipoprotein A (apo A): 0.19 g/L vs. 0.35 g/L; apolipoprotein B (apo B): 0.71 g/L vs. 1.00 g/L; high-density lipoprotein cholesterol (HDL-C): 0.15 mmol/L vs. 0.18 mmol/L; low-density lipoprotein cholesterol (LDL-C) : 0.77 mmol/L vs. 1.22 mmol/L. all *P* < 0.001). While serum lipid levels were significantly reduced in all ACLF patients, the reduction was greater in nonsurvivors. Thus, there was a clear tendency to lower serum lipid levels in line with the severity of the disease.

### Dyslipidemia as an important assessment indicator for patients with HEV-triggered ACLF

To further elucidate the relationship between dyslipidemia and disease severity in HEV-triggered ACLFs, we first examined whether there were differences between the presence and absence of BI. (Supplementary Fig. 1). We found that patients with BI were generally comparable to those without BI in terms of apo B and HDL-C levels, while other lipids (cholesterol, triacylglycerol, apoA and LDL-C) in cases of BI showed a significantly lower level (*P* < 0.05). Second, serum lipid concentrations in patients with HEV-triggered ACLF were analyzed and compared across groups with increasing organ failures (four subgroups: patients without, with 1, 2 or more than 3 organ failures). In fact, serum lipid levels in the four subgroups differed substantially. We found that the majority of serum lipids in HEV-triggered ACLF patients with ≥ three organ failures were significantly lower than those in other groups (*P* < 0.05), and serum lipid levels showed a decreasing trend with the increase in organ failures. (Supplementary Fig. 2). These findings suggest that serum lipids are implicated in the severity of the disease.

To understand the contributions of serum lipids to the short-term mortality without LT over 90 days, we further incorporated the well-known potential (age, cirrhosis, ascites, HE, Tbil and INR) and other possible factors (including serum lipid levels, platelet counts and others) into the multivariate Cox regression analysis. Our analysis revealed that HE and ascites were indeed the most important factors for short-term mortality (hazard ratios (HRs) = 2.027 and 1.855, *P* < 0.05). Additionally, to our surprise, two unexpected predictors (triacylglycerol and apoA) were identified as being associated with short-term mortality after adjusting for confounding factors in these ACLF patients (HR = 0.551, *P* < 0.05 and HR = 0.200, *P* < 0.05). (Table 1). Cirrhosis (*P* = 0.059), BI (*P* = 0.947), Tbil (*P* = 0.968) and INR (*P* = 0.079) were not significant contributors to short-term mortality. Moreover, associations between serum lipid levels and short-term mortality were corroborated by subgroup analysis (one with CHB etiology and one with fatty liver disease (FLD) etiology), which also identified the effect of dyslipidemia (Supplementary Tables 3 and Supplementary Table 4). In contrast, HBV infection may not significantly affect disease outcomes. Furthermore, the etiology of preexisting FLD did not confound the value of apoA in mortality prediction, and the level of triacylglycerol also showed a tendency of predictive potency (*P* = 0.065). Therefore, it was strongly suggested that dyslipidemia is associated with the pathophysiology of HEV-triggered ACLF.

### Novel scoring model for HEV-triggered ACLF

The above systemic analysis indicated that ascites, HE, apoA and triacylglycerol levels were significantly associated with short-term mortality in HEV-triggered ACLF patients. Therefore, we conducted a multivariate Cox PH analysis including the four major indicators noted above to design and calculate a new prognostic score (a detailed description is provided in Supplementary Table 5). The formula for this new scoring model is shown below:

HEV-T-ACLFs = 0.632×(ascites: no, 1 point; mild to moderate, 2 points; severe, 3 points) + 0.865×(HE: no, 1 point; grade 1–2, 2 points; grade 3–4, 3 points) − 0.413×triacylglycerol mmol/L) − 2.171×apoA (g/L).

**Fig. 2:**
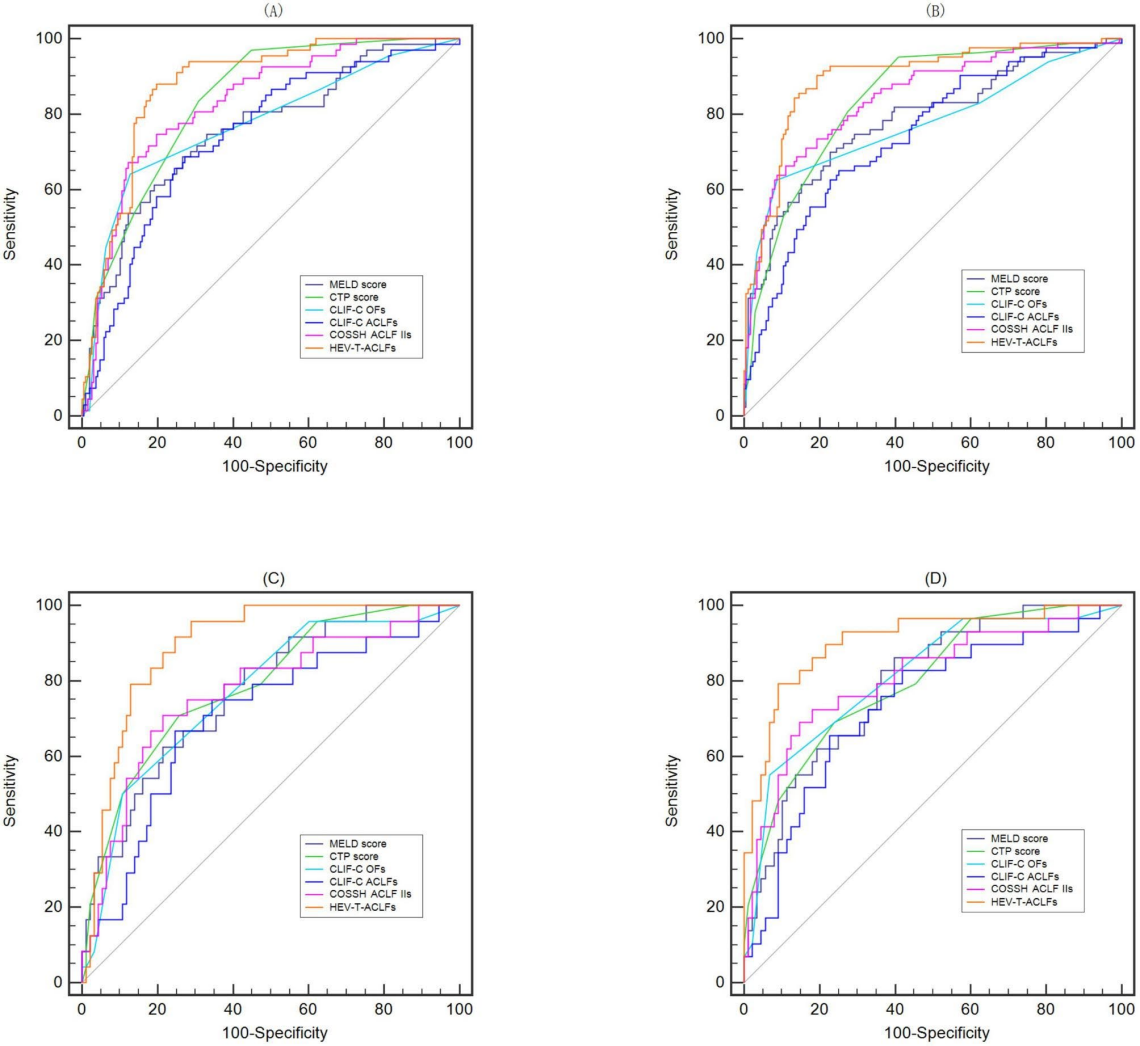
AUROCs of different prognostic models to predict 28-day and 90-day mortality in patients with HEV-triggered ACLF. (A-B) AUROC curves of these prognostic models for predicting the (**A**) 28-day and (**B**) 90-day mortality in the training set. (**C-D**) AUROCs curves of these prognostic models for predicting the (**C**) 28-day and (**D**) 90-day mortality in the test set **Abbreviations**: AUROC, area under the receiver operating characteristic curve; HEV, hepatitis E virus; ACLF, acute-on chronic liver failure; MELD score, model for end-stage liver disease score; CTP score, Child-Turcotte-Pugh score; CLIF-C OFs, Chronic Liver Failure-Consortium (CLIF) organ failure score; CLIF-C ACLFs, Chronic Liver Failure-Consortium acute-on chronic liver failure score; COSSH ACLF IIs, Chinese Group on the Study of Severe Hepatitis B acute-on chronic liver failure II score; HEV-T-ACLFs, hepatitis E virus triggered acute-on chronic liver failure score.

AUROC curve analysis was then conducted to assess and compare the novel HEV-T-ACLFs (Fig. 2; Table 3). Compared to the four previous well-known prognostic scoring systems (MELD score, CTP score, CLIF-C organ failure score (OFs) and CLIF-C ACLFs), the HEV-T-ACLFs showed a more accurate prognosis, with the highest auROCs predicting both 28-day and 90-day mortality in patients with HEV-triggered ACLF (auROCs were 0.878 and 0.896, respectively; *P* < 0.05). Table 3 shows the pairwise comparison of auROCs. Although compared to the COSSH ACLF IIs, the difference was not significant (*P* = 0.1075 and 0.0545), HEV-T-ACLFs had obvious advantages over the other four scoring systems (all *P* < 0.05). A subsequent test group analysis of 117 patients also confirmed a similar predictive value for the HEV-T-ACLFs (auROCs for 28-day and 90-day mortality without LT were 0.892 and 0.906, respectively). The performance of the HEV-T-ACLF score was similar to that of COSSH ACLF IIs for the prediction of 28-day and 90-day mortality (*P* > 0.05), while HEV-T-ACLFs had obvious advantages over the other four prognostic scoring systems (all *P* values < 0.05).

**Table 3:** AUROCs for predicting the 28-day and 90-day mortality of patients with HEV-triggered ACLF.

Models	For predicting the 28-day mortality			For predicting the 90-day mortality		
	AUROC (95% CI)	Z value	*P* value	AUROC (95% CI)	Z value	*P* value
**Training set (n = 254**)						
HEV-T-ACLFs	0.878 (0.833–0.922)			0.896 (0.853–0.939)		
MELD score	0.758 (0.689–0.827)	3.349	0.0008	0.784 (0.721–0.848)	3.467	0.0005
CTP score	0.836 (0.787–0.885)	2.153	0.0313	0.840 (0.790–0.890)	3.192	0.0014
CLIF-C OFs	0.774 (0.705–0.842)	3.061	0.0022	0.776 (0.710–0.842)	3.699	0.0002
CLIF-C ACLFs	0.742 (0.673–0.810)	3.625	0.0003	0.745 (0.681–0.810)	4.339	< 0.0001
COSSH ACLF IIs	0.831 (0.776–0.886)	1.610	0.1075	0.845 (0.792–0.898)	1.923	0.0545
**Test set (n = 117**)						
HEV-T-ACLFs	0.892 (0.833–0.952)			0.906 (0.840–0.973)		
MELD score	0.771 (0669-0.873)	2.232	0.0256	0.791 (0.702–0.881)	2.256	0.0241
CTP score	0.787 (0.688–0.887)	2.650	0.0080	0.799 (0.709–0.889)	2.888	0.0039
CLIF-C OFs	0.768 (0.672–0.864)	2.437	0.0148	0.813 (0.729–0.896)	1.948	0.0514
CLIF-C ACLFs	0.704 (0.583–0.862)	2.683	0.0073	0.735 (0.627–0.842)	2.667	0.0077
COSSH ACLF IIs	0.770 (0.658–0.882)	1.941	0.0523	0.808 (0.710–0.906)	1.739	0.0821

NOTE: AUROCs for different models were calculated and compared using the Z test (Delong’s method). The optimal cut-of points were determined by maximizing Youden index.

Abbreviations: AUROC, area under the receiver operating characteristic curve; HEV, hepatitis E virus; ACLF, acute-on chronic liver failure; HEV-T-ACLFs, HEV triggered ACLF score; MELD score, model for end-stage liver disease score; CTP score, Child-Turcotte-Pugh score; CLIF-C OFs, Chronic Liver Failure-Consortium (CLIF) organ failure score; COSSH ACLF IIs, Chinese Group on the Study of Severe Hepatitis B acute-on chronic liver failure II score.

## Discussion

To date, there has been no pathophysiology-based, noninvasive predictive model that can accurately evaluate the prognosis of HEV-triggered ACLF. Furthermore, the existing noninvasive ACLF scoring systems are likely unsuitable because of different etiologies and disease progression. Here, we investigated the record number of patients infected with HEV over an 11-year period for analysis in this study. As mentioned above, by screening a large number of hospitalized patients infected with HEV, we were able to study in depth the characteristics of abnormal clinical factors in HEV-triggered ACLFs. Through comparative analysis, we confirmed and expanded current data on the clinical characteristics of HEV-triggered ACLF. In particular, a simple and accurate prognostic scoring model for HEV-triggered ACLF was established.

First, in line with our previous and other reports [21–23] on the role of CLDs in HEV infections, the short-term mortality rate for HEV-triggered ACLF (approximately 30% in this study) was indeed high. In our study, a number of clinical management indicators were considered in assessing liver function and disease severity for HEV-triggered ACLF. In addition to more severe liver dysfunction and coagulopathy disorder, other special findings were the unusually low serum lipid levels in patients with ACLF and patients with short-term mortality. These specific clinical features suggest that HEV superinfection may have different biochemical abnormalities. Although there is no direct evidence of serum lipid inhibition as a result of HEV infection to date, the influence of lipid homeostasis on HEV infection has been highlighted by a number of previous studies [16, 17]. Furthermore, as is well known, one of the most important bases for assessing the severity of liver failure is systemic inflammatory response syndrome [1, 24]. This means that bacterial infection is very important for ACLF. Here, for the first time, we found a strong correlation between BI and decreased levels of serum lipids. In retrospect, we think that the data align very well with the pathophysiology of lipid metabolism and ACLF. The liver serves as an energy reservoir and hub of metabolism, which stores lipids (such as triglycerides, cholesterol, etc.) and polysaccharides and releases them during the course of high energy consumption in the body [25]. Imbalances in lipid homeostasis present in the pathological condition caused by severe liver decompensation may lead to excessive accumulation of lipids in hepatocytes, known as hepatic steatosis [10, 26]. We believe this may be one of the main causes of lipid redistribution due to the accumulation of fat droplets in the liver and a marked decrease in serum lipids. On the other hand, systemic inflammation is a distinctive feature of ACLF and may induce significant metabolic changes and seriously affect lipid metabolism [25, 26].

Second, it is well known that organ failure is the most representative clinical characteristic to evaluate the stage and severity of liver failure [9, 27, 28]. In recent years, appreciation of the complexity of organ failure has increased considerably, and researchers have realized that imbalances in multiple organ failure are particularly associated with poor prognosis. In this study, it was surprising to find that dyslipidemia was closely related to various organ failures in HEV-triggered ACLFs. In addition to the presence of well-known risk factors, such as ascites and HE, our data indicate that lipid components, including low concentrations of apoA and triglycerides, are associated with short-term mortality. We postulated that the reason for the unexpected decrease in triglycerides and apoA could be caused by the severe immune metabolic disorder mentioned above. We then questioned whether the etiologies of CHB and FLD on HEV-triggered ACLF could affect the prognostic value of serum lipids. However, the subgroup analysis reached similar conclusions regarding serum lipids as independent risk factors for short-term mortality while incorporating other confounding factors. These data reveal two new insights. First, factors related to HBV infection (viral load, viral markers and antiviral therapy status) may not significantly affect disease outcomes in HEV-triggered ACLF. Second, dyslipidemia remains a risk factor for predicting poor prognosis in HEV-ACLF independently of basal lipid metabolism. As a result, we strongly believe that dyslipidemia has exacerbated the clinical outcomes of HEV-triggered ACLF.

Third, the clinical characteristics of serum lipids indicate that an appropriate new scoring model containing dyslipidemia may be helpful in accurately assessing the prognosis of HEV-triggered ACLF. The new scoring model was found to be more accurate in the assessment of LT-free short-term mortality than the previous four generic scoring models (MELD score, CTP score, CLIF-C OFs and CLIF-C ACLFs) [9]. Furthermore, although the new model was similar in predictive potency to COSSH ACLF IIs, the HEV-T-ACLFs are certainly simpler with parameters easily accessible in clinical practice. Detection of serum lipids is inexpensive and easy to assess, indicating that our new HEV-triggered ACLF scoring model simplifies existing predictive models. Furthermore, the subgroup analysis (with CHB or FLD etiologies) and the test cohort analysis strongly support the prognostic accuracy of this new model. In addition, regardless of whether cirrhosis is present, this new scoring model accurately predicts clinical outcomes. Importantly, this new scoring model fills a gap in predictive models specific to ACLF triggered by HEV infection. However, this does not mean that dyslipidemia is related only to the disease caused by HEV, as previous studies have shown that lipids or their metabolic disorder were associated with stratification of severe hepatitis diseases in other cohorts [15, 29, 30] and have indicated poor prognosis [31, 32]. In contrast, dyslipidemia may be the common pathophysiological response of ACLF. Despite not being applied to clinical practice, our data may suggest its general value for clinical prediction models for ACLF triggered by other causes as well.

### Comparisons with other studies and what does the current work add to the existing knowledge

On the one hand, the HEV-T-ACLFs are simpler with parameters easily accessible in clinical practice compared to existing generic scoring models. Detection of serum lipids is inexpensive and easy to assess, indicating that our new HEV-triggered ACLF scoring model simplifies existing predictive models. On the other hand, the new HEV-T-ACLFs also showed better predictive performance.

### Strengths and limitations

By placing this new scoring model into a clinical perspective, it can improve clinicians’ ability to predict disease and benefit the majority of patients. However, due to the limitations of this study, further investigation of the causes of dyslipidemia in HEV-triggered ACLF was not conducted. It is therefore necessary to further validate the results and explore the underlying mechanism.

## Conclusions

Predicting short-term mortality in HEV-triggered ACLF is not only critical for proper prognosis judgment but also important in determining a pathogenic link to identify potential intervention strategies. This study is among the first to discuss the correlation between hepatitis E and serum lipids and provides a new, simple and accurate predictive scoring model for HEV-triggered ACLF.

## Electronic supplementary material

Below is the link to the electronic supplementary material.

Supplementary Material 1

## Data Availability

The data can be obtained from the corresponding author upon reasonable request.

## Abbreviations

ACLF: Acute-on-chronic liver failure
ALT: Alanine aminotransferase
APASL: Asian Pacific Association for the Study of the Liver
apoA: apolipoprotein A
apoB: apolipoprotein B
AST: Aspartate aminotransferase
AUROC: Areas under receiver operator characteristic
BI: Bacterial infection
CHB: Chronic hepatitis B
CLDs: Chronic liver diseases
CLIF-C OFs: Chronic Liver Failure-Consortium organ failure score
CLIF-C: Chronic Liver Failure-Consortium
CLIF-SOFA: Chronic Liver Failure-Sequential Organ Failure Assessment
COSSH: Chinese Group on the Study of Severe Hepatitis B
CTP: Child-Turcotte-Pugh
Dbil: Direct bilirubin
FLD: Fatty liver disease
HBV: Hepatitis B virus
HCV: Hepatitis C virus
HDL-C: High-density lipoprotein cholesterol
HE: Hepatic encephalopathy
HEV: Hepatitis E virus
HR: Hazard rates
INR: International Normalized Ratio
LDL-C: Low-density lipoprotein cholesterol
LT: Liver transplantation
MELD: Model for End-Stage Liver Disease
PTA: Prothrombin activity
TBA: Total bile acid
Tbil: Total bilirubin
WBC: White blood cells
