# Supplementary material for: A new dyslipidemia-based scoring model to predict transplant-free survival in patients with hepatitis E-triggered acute-on-chronic liver failure
Source: Lipids Health Dis. 2023 Jun 24;22:80. doi: 10.1186/s12944-023-01826-y (PMC10290386; doi:10.1186/s12944-023-01826-y)
Supplement: Supplementary file 1 — Supplementary Material 1 [file 12944_2023_1826_MOESM1_ESM.pdf]

Supplementary Materials

A new dyslipidemia-based scoring model to predict transplant-free survival in patients with hepatitis E-triggered acute-on-chronic liver failure

Chong Chen[1, †], Aihong Zhu[1, †], Shanke Ye[1, †], Weixia Li[1], Ling Fei[2], Qin Huang[1, *], Liang Chen[2, *]

[1]Department of Infectious Diseases, Shanghai Public Health Clinical Center, Fudan University, Shanghai 201508, China;

[2]Department of Hepatology, Shanghai Public Health Clinical Center, Fudan University, Shanghai 201508, China.

[†]Chong Chen, Aihong Zhu and Shanke Ye contributed equally to this work.

***Corresponding Authors:** Prof. Liang Chen and Prof. Qin Huang.

Shanghai Public Health Clinical Center, Fudan University, 2901 Caolang Road, Jin-Shan District, Shanghai 201508, P.R. China

E-mail: chenliang@shaphc.org and huangqin@shaphc.org

Tel: +86(21)57248785;

Fax: +86(21)57248782.

List of Supplemental Digital Content

Supplementary Files	
**Supplementary File 1**	The inclusion of and exclusion criteria for HEV-triggered ACLF.
**Supplementary File 2**	Laboratory detection and instruments used in this study.
**Supplementary File 3**	Diagnosis of HEV infection, liver cirrhosis, and ACLF.
**Supplementary File 4**	Definition and range of underlying chronic liver diseases.
Supplementary Tables	
**Supplementary Table 1**	The number and prevalence of organ failure in patients with HEV-triggered ACLF upon admission.
**Supplementary Table 2**	Baseline characteristics and outcome of patients with HEV-triggered ACLF in the training and test cohorts.
**Supplementary Table 3**	Risk factors associated with 90-day mortality in patients with CHB etiology.
**Supplementary Table 4**	Risk factors associated with 90-day mortality in patients with fatty liver disease etiology.
**Supplementary Table 5**	Risk factors associated with 90-day mortality in patients from the training cohort using a multivariate Cox PH Model.
Supplementary Figures	

Supplementary Figure 1	Serum lipid levels between patients with or without bacterial infection among HEV-triggered ACLFs at the time of study enrollment.
Supplementary Figure 2	Baseline serum lipid levels in HEV-triggered ACLFs with no, one, two or ≥ three organ failures.

Supplementary File 1

The inclusion of and exclusion criteria for HEV-triggered ACLF.

4952 patients infected with HEV were initially identified. A total of 3427 cases were then excluded for several reasons: 2843 outpatients, 222 pregnancy women, 118 for lack of medical records, 66 for HIV co-infections, 87 for having tuberculosis, 10 for coexistence of other viral infection markers (including 5 anti-HBc IgM+ individuals, 3 HAV IgM+ individuals, 1 CMV IgM+ individual and 1 CMV/EBV IgM+ individual.) and 19 duplicate cases. Furthermore, patients who received lipid-lowering therapy (n=62) were also excluded from initial screening. Seven patients received liver transplantation within 90 days of the diagnosis of ACLF and were excluded; the remaining 817 patients were therefore included in the final analysis.

Supplementary File 2

Laboratory detection and instruments used in this study.

In this study, anti-HEV immunoglobulin M (IgM) and G (IgG) level were tested using enzyme-linked immunosorbent assay (ELISA) (MP Biomedicals, Singapore). Liver function and serum lipids were examined by an automated biochemistry-analysis instrument (7600 Series; Hitachi, Japan). Coagulation indicators were measured by an automatic coagulometer (STA-R; Diagnostica Stago, Asnieres-sur-Seine, France), and the international normalized ratio (INR) was calculated as well. Serum HBV markers were detected using ELISA (ARCHITECT i2000 SR; Abbott, Germany). HBV DNA quantification was detected using real-time PCR (ABI 7500; Applied Biosystems Inc, United States).

Supplementary File 3

Diagnosis of HEV infection, liver cirrhosis, and ACLF.

HEV infection was diagnosed by two consecutive positive serum anti-HEV immunoglobulin M (IgM) test and by seroconversion to positive HEV immunoglobulin G during follow-up recorded in the hospital clinical database.

Liver cirrhosis was defined histologically as a form of distortion of the hepatic architecture and regenerative nodule, which was confirmed by radiological or histological evidence[1].

ACLF was defined according to Asian Pacific association for the study of the liver (APASL) criterion: an acute hepatic insult manifesting as jaundice (serum bilirubin ≥ 5 mg/dL (85 micromol/L) and coagulopathy (INR ≥ 1.5 or prothrombin activity < 40%) complicated within 4 weeks by clinical ascites and/or encephalopathy in a patient with previously diagnosed or undiagnosed chronic liver disease/cirrhosis[2].

References

1 Lefton HB, Rosa A, Cohen M. Diagnosis and epidemiology of cirrhosis. *Med Clin North Am* 2009; 93: 787-99.

2 Sarin SK, Choudhury A, Sharma MK, et al. Acute-on-chronic liver failure: consensus recommendations of the Asian Pacific association for the study of the liver (APASL): an update. *HEPATOL INT* 2019; 13: 353-90.

Supplementary File 4

Definition and range of underlying chronic liver diseases.

The definition of underlying chronic liver diseases was the presence of one or more of the following diseases: primary hepatic carcinoma, chronic hepatitis B, chronic hepatitis C, fatty liver disease, alcoholic liver disease, autoimmune liver disease, schistosomiasis or cirrhosis with unknown factor.

Supplementary Table 1. The number and prevalence of organ failure in patients with HEV-triggered ACLF upon admission.

Organ failure	Number of patients	Prevalence (%)
**Total HEV triggered ACLFs (n=371)**		
Liver	222	59.8
Coagulation	45	12.1
Kidney	14	3.8
Brain	9	2.4
Circulatory	1	0.3
Respiratory	1	0.3
**Training set (n=254)**		
Liver	157	61.8
Coagulation	34	13.4
Kidney	7	2.8
Brain	6	2.4
Circulatory	1	0.4
Respiratory	1	0.4
**Test set (n=117)**		
Liver	65	55.6
Coagulation	11	9.4
Kidney	7	6.0
Brain	3	2.6

Circulatory	0	0
Respiratory	0	0

NOTE: Organ failure was assessed at admission in this table. Liver failure: Tbil ≥ 12mg/dL. Coagulation failure: INR > 2.5. Kidney failure: serum creatinine ≥ 2mg/dL. Brain failure: HE grade III or IV. Circulatory failure: using vasoactive agents. Respiratory failure: a ratio of PaO2 of arterial oxygen to FiO2 of ≤ 200 or an SpO2 to FiO2 ratio of ≤ 200.

Abbreviations: HEV, hepatitis E virus; ACLF, acute-on-chronic liver failure; Tbil, total bilirubin; INR, international normalized ratio; HE, hepatic encephalopathy.

Supplementary Table 2. Baseline characteristics and outcome of patients with HEV-triggered ACLF in the training and test cohorts.

Patients' variable	Training set (n=254)	Test set (n=117)	*P* value	Total patients (n=371)
Basic characteristics				
Age (y)	54 (43-63)	60 (54-67)	0.001	55 ± 14
Gender (male, %)	206 (81.1)	102 (87.2)	0.182	308 (83.0)
Cirrhosis, n (%)	143 (56.3)	48 (41.0)	0.007	191 (51.5)
Prognostic scores (continuous)				
MELD score	17 (10-21)	14 (9-18)	0.132	15 (10-20)
CTP score	9 (7-10)	9 (7-10)	0.266	9 (7-10)
CLIF-C OFs	8 (7-9)	8 (7-8)	0.036	8 (7-8)
CLIF-C ACLFs	40 (34-44)	42 (36-46)	0.233	41 (34-45)
COSSH ACLF IIs	7 (6-8)	6 (6-7)	< 0.001	7 (6-7)
Date used to create new score				
Ascites, n (%)	92 (36.2)	57 (48.7)	0.030	149 (40.2)
Hepatic encephalopathy, n (%)	40 (15.8)	9 (7.7)	0.033	49 (13.2)
Triacylglycerol, mmol/L	1.46 (1.00-2.15)	1.90 (1.27-2.97)	< 0.001	1.56 (1.10-2.36)
ApoA, g/L	0.26 (0.13-0.53)	0.30 (0.20-0.48)	0.176	0.27 (0.15-0.50)
Clinical organ failure, n (%)				
Liver	185 (72.8)	85 (72.7)	NA	270 (72.8)
Coagulation	91 (35.8)	33 (28.2)	0.157	124 (33.4)

Kidney	17 (6.7)	7 (6.0)	NA	24 (64.7)
Brain	39 (15.4)	7 (6.0)	0.011	46 (12.4)
Circulatory	7 (2.8)	2 (1.7)	0.725	9 (2.4)
Respiratory	1 (0.4)	0	NA	1 (0.3)
LT-free short-term mortality, n (%)				
28-day mortality	67 (26.4)	24 (20.5)	0.244	91 (24.5)
90-day mortality	83 (32.7)	29 (24.8)	0.056	112 (30.2)

NOTE: Data are presented as the median (Q1-Q3), the mean ± SD or the number of patients (%). The categorical data between two groups were compared with chi-square test or Fisher's exact test. The continuous data between two groups were compared by t test or Mann-Whitney test. Liver failure: Tbil ≥ 12mg/dL. Coagulation failure: INR > 2.5. Kidney failure: serum creatinine ≥ 2mg/dL. Brain failure: HE grade III or IV. Circulatory failure: using vasoactive agents. Respiratory failure: a ratio of PaO2 of arterial oxygen to FiO2 of ≤200 or an SpO2 to FiO2 ratio of ≤ 200.

Abbreviations: CLDs, chronic liver diseases; MELD score, model for end-stage liver disease score; CTP score, Child-Turcotte-Pugh score; CLIF-C OFs, Chronic Liver Failure-Consortium (CLIF) organ failure score; ACLFs, acute-on chronic liver failure score; COSSH ACLF IIs, Chinese Group on the Study of Severe Hepatitis B acute-on chronic liver failure II score; ApoA, apolipoprotein A; NA, not available; Tbil, total bilirubin; INR, international normalized ratio; HE, hepatic encephalopathy.

Supplementary table 3. Risk factors associated with 90-day mortality in patients with CHB etiology.

Parameters	Univariate analysis		Multivariate analysis	
	HR (95% CI)	*P* value	HR (95 % CI)	*P* value
Age, (y)	1.024 (0.999-1.050)	0.065		
Gender (male)	0.955 (0.370-2.460)	0.923		
Alcohol use	1.203 (0.619-2.339)	0.586		
Cirrhosis	1.897 (0.911-3.949)	0.087		
HBeAg positivity	0.325 (0.115-0.920)	< 0.05	0.412 (0.138-1.226)	0.111
HBV DNA (log10 copies/ml)	1.000 (1.000-1.000)	0.738		
Anti-HBV therapy	0.438 (0.134-1.429)	0.171		
Ascites	3.069 (1.582-5.956)	< 0.01	1.898 (1.002-3.986)	< 0.05
Bacterial infection	1.615 (0.757-3.447)	0.215		
Hepatic encephalopathy	4.031 (2.064-7.874)	< 0.01	2.176 (1.031-5.137)	< 0.05
Gastrointestinal haemorrhage	1.371 (0.765-2.855)	0.543		
ALT, U/L	NA	0.802		
AST, U/L	1.000 (1.000-1.001)	0.264		
Tbil, μmol/L	1.001 (1.000-1.002)	0.021	0.999 (0.997-1.001)	0.301
Albumin, g/L	0.966 (0.910-1.025)	0.252		
INR	1.551 (1.177-2.044)	0.002	1.156 (0.827-1.616)	0.397
Creatinine, μmol/L	1.003 (1.000-1.007)	0.066		
Glucose, mmol/L	1.025 (0.901-1.167)	0.705		

WBC, 10^9/L	1.074 (0.982-1.173)	0.116		
Haemoglobin, g/L	0.994 (0.983-1.006)	0.343		
Platelet, 10^9/L	0.997 (0.990-1.004)	0.383		
Cholesterol, mmol/L	0.485 (0.324-0.726)	< 0.001	1.067 (0.631-1.804)	0.810
Triacylglycerol, mmol/L	0.239 (0.125-0.457)	< 0.001	0.391 (0.135-0.967)	< 0.05
ApoA, g/L	0.024 (0.002-0.257)	0.002	0.059 (0.004-0.884)	< 0.05
ApoB, g/L	0.219 (0.080-0.602)	0.003	0.644 (0.129-3.218)	0.592
HDL-C, mmol/L	0.306 (0.070-1.346)	0.117		
LDL-C, mmol/L	0.700 (0.448-1.096)	0.119		

NOTE: Univariate and Multivariate Cox regression models were used to assess the associations between various risk factors and different disease outcomes, as indicated.

Abbreviations: LT, liver transplantation; HEV, hepatitis E virus; ACLF, acute-on-chronic liver failure; CHB, chronic hepatitis B; HR, hazard ratio; ALT, alanine aminotransferase; AST, aspartate aminotransferase; Tbil, total bilirubin; INR, international normalized ratio; WBC, white blood cell; ApoA, apolipoprotein A; ApoB, apolipoprotein B; HDL-C, high-density lipoprotein cholesterol; LDL-C, low-density lipoprotein cholesterol; NA, not available.

Supplementary table 4. Risk factors associated with 90-day mortality in patients with fatty liver disease etiology.

Parameters	Univariate analysis		Multivariate analysis	
	HR (95% CI)	*P* value	HR (95 % CI)	*P* value
Age, (y)	1.034 (0.985-1.086)	0.181		
Gender (male)	0.786 (0.174-3.545)	0.754		
Alcohol use	0.593 (0.182-1.924)	0.384		
Cirrhosis	2.875 (0.941-8.788)	0.064		
Ascites	1.708 (0.558-5.222)	0.348		
Bacterial infection	2.557 (0.836-7.816)	0.100		
Hepatic encephalopathy	4.375 (1.431-13.373)	< 0.05	2.865 (1.210-6.222)	< 0.05
Gastrointestinal haemorrhage	0.048 (0.000-126.74)	0.746		
ALT, U/L	NA	0.545		
AST, U/L	NA	0.876		
Tbil, μmol/L	1.001 (0.998-1.004)	0.477		
Albumin, g/L	0.911 (0.809-1.025)	0.121		
INR	1.490 (1.105-2.008)	< 0.01	1.101 (0.767-1.519)	0.477
Creatinine, μmol/L	1.000 (0.986-1.014)	0.972		
Glucose, mmol/L	0.887 (0.720-1.094)	0.263		
WBC, 10^9/L	1.066 (1.004-1.131)	< 0.05	1.001 (1.000-1.001)	0.865
Haemoglobin, g/L	0.989 (0.956-1.023)	0.527		
Platelet, 10^9/L	0.991 (0.980-1.002)	0.106		

Cholesterol, mmol/L	0.387 (0.200-0.752)	< 0.01		
Triacylglycerol, mmol/L	0.603 (0.291-0.876)	< 0.05	0.832 (0.463-1.123)	0.065
ApoA, g/L	0.003 (0.000-0.231)	< 0.01	0.121 (0.089-0.356)	< 0.05
ApoB, g/L	0.400 (0.096-1.663)	0.207		
HDL-C, mmol/L	0.311 (0.020-4.876)	0.405		
LDL-C, mmol/L	0.406 (0.179-0.924)	< 0.05	0.998 (0.865-1.101)	0.654

NOTE: Univariate and Multivariate Cox regression models were used to assess the associations between various risk factors and different disease outcomes, as indicated.

Abbreviations: LT, liver transplantation; HEV, hepatitis E virus; ACLF, acute-on-chronic liver failure; HR, hazard ratio; ALT, alanine aminotransferase; AST, aspartate aminotransferase; Tbil, total bilirubin; INR, international normalized ratio; WBC, white blood cell; ApoA, apolipoprotein A; ApoB, apolipoprotein B; HDL-C, high-density lipoprotein cholesterol; LDL-C, low-density lipoprotein cholesterol; NA, not available.

Supplementary table 5. Risk factors associated with 90-day mortality in patients from the training cohort using a multivariate Cox PH Model.

	**Regression coefficient**	**HR (95%CI)**	***P* value**
Ascites	0.632	1.881 (1.197-2.955)	0.006
Hepatic encephalopathy	0.865	2.375 (1.509-3.738)	< 0.001
Triacylglycerol, mmol/L	-0.413	0.662 (0.473-0.925)	0.016
ApoA, g/L	-2.171	0.114 (0.037-0.356)	< 0.001

Abbreviations: LT, liver transplantation; HEV, hepatitis E virus; ACLF, acute-on-chronic liver failure; HR, hazard ratio; ApoA, apolipoprotein A.

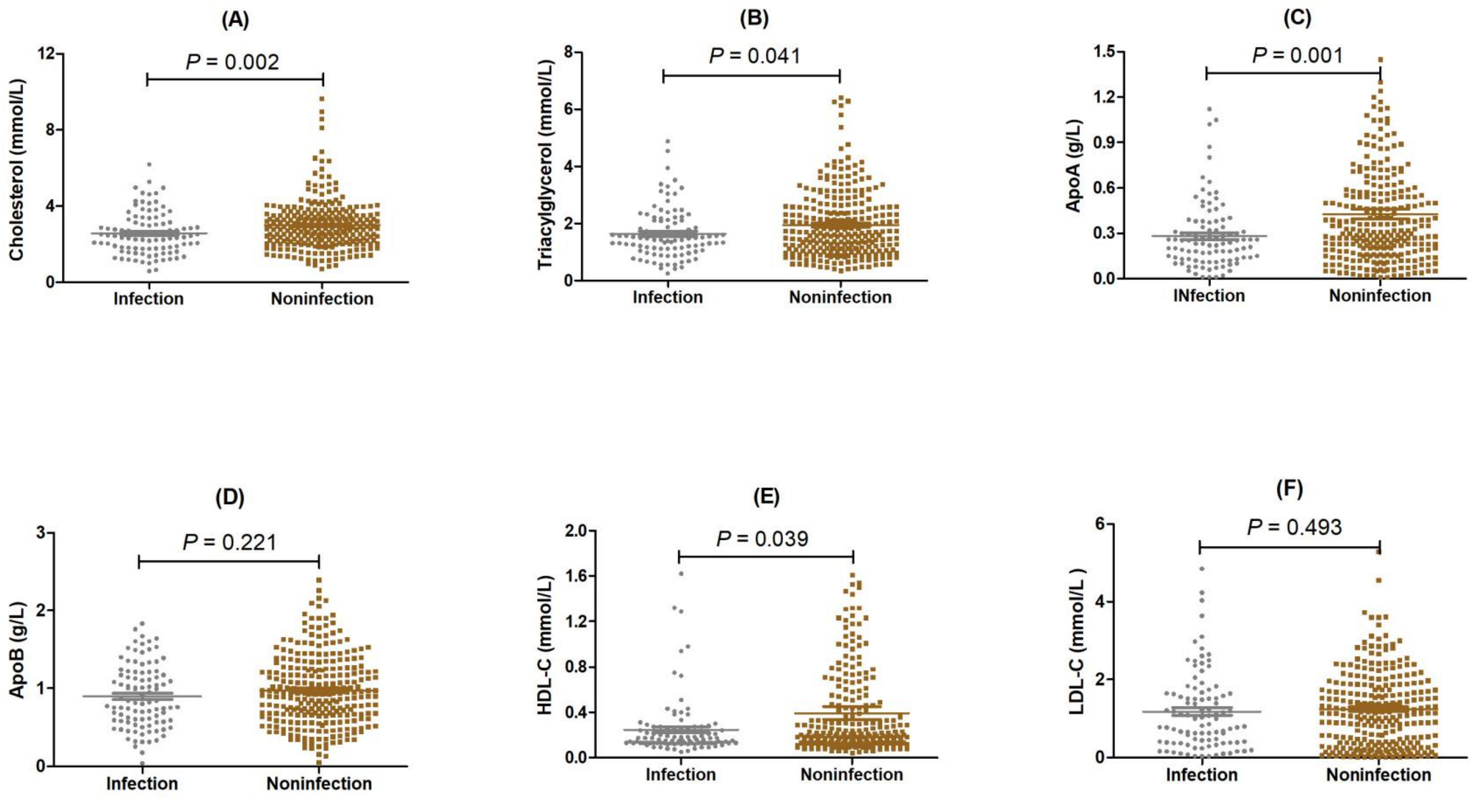

Supplementary Figure 1. Serum lipid levels between patients with or without bacterial infection among HEV-triggered ACLFs at the time of study enrollment.

Abbreviations: ApoA, apolipoprotein A; ApoB, apolipoprotein B; HDL-C, high-density lipoprotein cholesterol; LDL-C, low-density lipoprotein cholesterol.

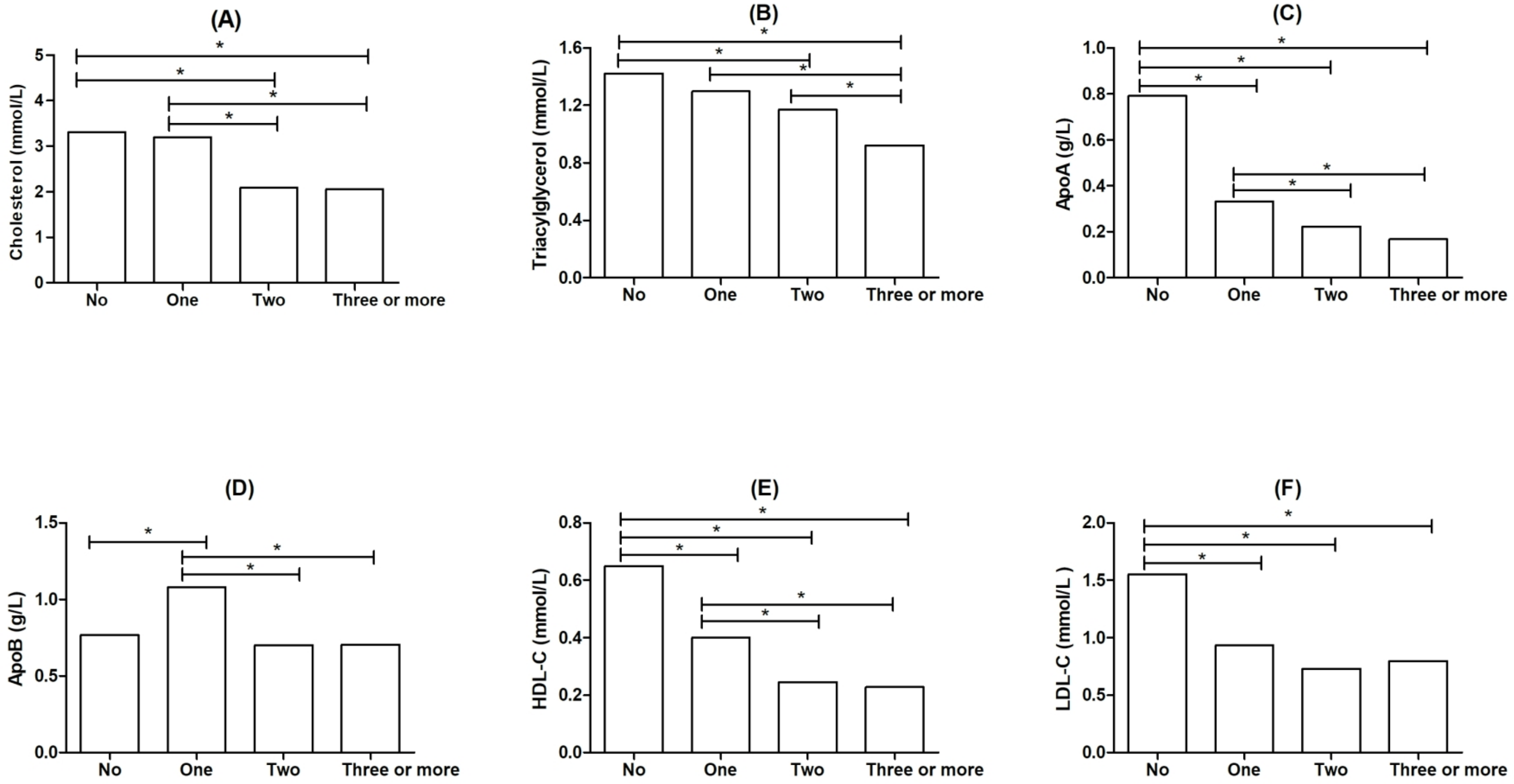

Supplementary Figure 2. Baseline serum lipid levels in HEV-triggered ACLFs with no, one, two or ≥ three organ failures.

Organ failure was assessed within 90 days after admission. Liver failure criteria: Tbil ≥ 12mg/dL. Coagulation failure criteria: INR > 2.5. Kidney failure:

serum creatinine ≥ 2mg/dL. Brain failure criteria: HE grade III or IV. Circulatory failure criteria: using vasoactive agents. Respiratory failure criteria: a ratio of PaO2 of arterial oxygen to FiO2 of ≤ 200 or an SpO2 to FiO2 ratio of ≤ 200.

Abbreviations: ApoA, apolipoprotein A; ApoB, apolipoprotein B; HDL-C, high-density lipoprotein cholesterol; LDL-C, low-density lipoprotein cholesterol.
